# Development and characterization of a fiber optical fluorescence sensor for the online monitoring of biofilms and their microenvironment

**DOI:** 10.1002/elsc.201900140

**Published:** 2020-03-02

**Authors:** Jana Schlaugat, Kai Patzer, Thorleif Hentrop, Dörte Solle, Iliyana Pepelanova, Uwe Schröder, Thomas Scheper

**Affiliations:** ^1^ Institute of Technical Chemistry Leibniz University Hannover Hannover Germany; ^2^ Institute of Environmental and Sustainable Chemistry Technische Universität Braunschweig Braunschweig Germany

**Keywords:** biofilm analysis, biogenic fluorophores, fluorescence sensor, online monitoring

## Abstract

The growth of microorganisms on surfaces and interfaces as a biofilm is very common and plays important role in various areas such as material science, biomedicine, or waste treatment among others. Due to their inhomogeneous structure and the variance in the microorganism consortium, the analysis of biofilms represents a significant challenge. An online fluorescence sensor was developed that is able to measure the most important biological fluorophores (proteins, nicotinamide adenine dinucleotide, and flavin) in a noninvasive manner in biofilms, e.g. in bioelectrochemical applications. The sensor gives the opportunity to continuously draw conclusions on the metabolic state of the biofilm. The developed sensor has a diameter of 1 mm at the sensor tip and can be moved on and into the biofilm surface. In the first experiment, the measuring range of the sensor and the long‐term stability could be determined and the system applicability was confirmed. In addition, measurements in biofilm‐like structures could be performed. The formation of a wastewater‐based biofilm was monitored using the developed sensor, demonstrating the functionality of the sensor in a proof‐of‐principle experiment.

**Abbreviations**
NADH: Nicotinamide adenine dinucleotide
RFI: relative fluorescence intensity

## 1 INTRODUCTION

Glass fiber–based fluorescence sensors are widely used in bioprocess technology for online monitoring of substrate and product concentrations [1, 2, 3, 4, 5, 6, 7]. They enable a highly sensitive and noninvasive measurement of various parameters during cultivation processes. Fluorescence sensors can be used to measure biogenic fluorophores like nicotinamide adenine dinucleotide (NADH), riboflavin, and proteins to draw conclusions about the metabolic state of the microorganisms [8, 9, 10]. Depending on the metabolite to be analyzed, different wavelengths are selected for excitation and emission [11].

Optical fluorescence sensors for biogenic fluorophores are currently not established for the measurement and analysis of microbial biofilms [12]. The challenge of analyzing a biofilm is its inhomogeneous and constantly changing structure and composition [13, 14, 15]. Biofilms are single species or mixed cultures, whose bacteria are embedded in a matrix of self‐produced extracellular polymeric substances, which allows the microorganisms to adjust to surrounding conditions and protects the colonies against environmental influences [16]. The composition of the matrix can be modified in response to changing environmental conditions by the cells in the biofilm. Therefore, a biofilm possesses the ability to adapt itself to its microenvironment and is constantly in a state of flux [17]. On the one hand, this flexibility makes the biofilm robust. On the other hand, it makes the analysis of biofilms a complicated and complex procedure. At the moment, the analysis of a biofilm is performed by interruption of the cultivation process. Typical methods focus on the study of adhesion between microorganisms and surface by using atomic force microscopy. Biofilms are also frequently analyzed concerning the composition of microorganisms and extracellular polymeric substances. For this purpose, methods like fluorescence in situ hybridization, scanning electron microscopy, and confocal laser scanning microscopy are usually used. The disadvantage of current biofilm analysis methods is that they can only be used to follow dynamic biofilm changes during cultivation in a limited way. Confocal laser scanning microscopy can be used for analyzing dynamic changes, but only in a flow cell where the biofilm grows on a transparent surface [15]. A brief overview of methods for analyzing the chemical profile of biofilms is presented in Table 1.

**PRACTICAL APPLICATION**

The newly developed sensor enables an online analysis of biofilms based on the monitoring of proteins, NADH, and flavin. These components give an insight into the metabolism of the biofilm. The sensor has a measuring tip with a diameter of 1 mm that allows measurements with a high resolution. Therefore, the sensor is of great interest for getting a better understanding of biofilm growth. Practical applications of the new sensor can be the analysis of a biofilm, e.g. in a microbial fuel cell, in pipelines, or other biofilm‐based processes, especially in cases where biofilms cannot be analyzed with established methods like confocal laser scanning microscopy.

**TABLE 1:** Brief overview of some methods for analyzing biofilm structure regarding their chemical profile [18, 19]

Method	Application	Advantages	Disadvantages	Reference
Confocal laser scanning microscopy	-3D analysis -Often used in combination with markers to analyze specific structures	-Sharp images -3D images	-Experience required -Expensive device	[20, 21]
Atomic force microscopy	-Quantitative biofilm analysis -Determination of adhesion forces	-Nondestructive technique -Works under ambient conditions	-Large area survey scan not possible -Sample damage or artifacts caused by tip shape and size -Expensive device and trained person needed	[22]
Infrared and Raman spectroscopy	-Chemical information	-In situ analysis	-Limited to surface spatial chemical changes	[23, 24]
Fluorescence in situ hybridization	-Phylogenetic information	-Can be combined with other methods to increase specificity	-Fluorescent labeling necessary	[20, 25, 26]
Fluorescent dyes and proteins	-Analyze biofilm features	-Large selection of dyes and proteins for several structures available	-Preparation of cells necessary	[27, 28]

Some optical sensors are already being used for the analysis of biofilms. Usually, the focus has been on using turbidity sensors for the analysis of biofilm thickness and biomass [29, 30, 31]. Optical fluorescence–based microsensors were also used for the detection of yellow fluorescent protein produced by *Staphylococcus aureus* grown as a biofilm [32]. So far, optical fluorescence sensors have not been used for online measurements of metabolic changes in biofilms based on biogenic fluorophores, although they are successfully used in suspension cell cultures. Fluorescence sensors can enable a time‐dependent, as well as location‐dependent detection of a biofilm. To achieve this task, a fluorescence sensor must measure reliably both in and near the biofilm. Furthermore, the sensor should be mobile or multiplexable so that it can be used at different positions in the biofilm, thus reacting to structural changes [33].

In this work, a fluorescence sensor for biofilms was developed. The sensor setup is similar to a sensor developed by König et al., which was developed for monitoring bioprocesses of suspensions cells [34]. A major difference of the sensor in this work is the minimal diameter of the light guide that enables the measurement of multiple process variables simultaneously. In contrast to König et al. and similar conventional bioprocess fluorescence sensors, the developed sensor in this work possesses a smaller excitation and detection area and contains movable optical components. This is the only way to ensure the excitation and detection of several parameters via a single light guide with a small diameter. In addition, the sensor is mobile to ensure spatial resolution of the biofilm surface. Only by including mobility and movable optical components in the sensor design is it possible to obtain a meaningful and continuous assessment of biofilm metabolism and activity.

## 2 MATERIALS AND METHODS

### 2.1 Sensor setup

**FIGURE 1:**
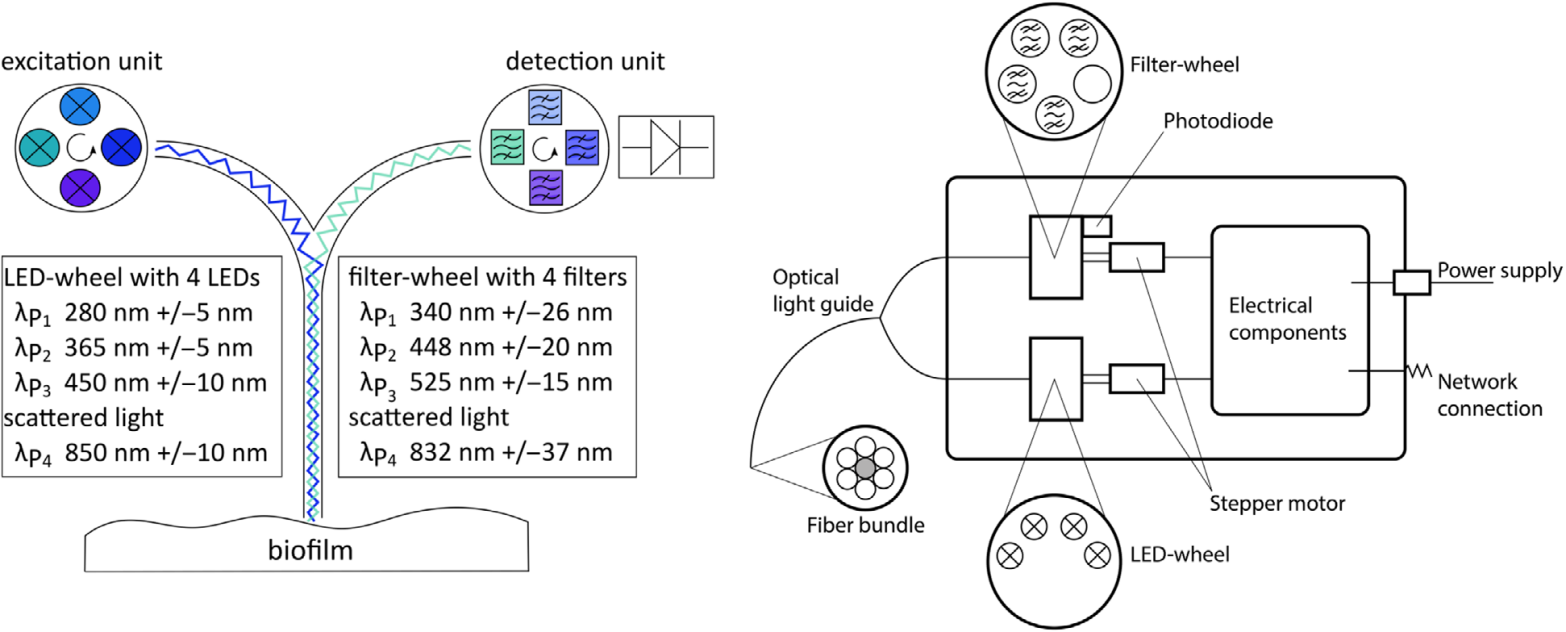
Left: Schematic structure of the developed sensor. The excitation unit and the detection unit are connected with the light guide. Wavelength of included LEDs and filters are listed. Right: Schematic illustration of the sensor box containing optical and electronic components

The developed sensor consists of an excitation unit, a detection unit, and a light guide. The light guide (art photonics GmbH, Germany) consists of seven silica fibers, one for excitation and six for emission. All fibers have a diameter of 200 µm and the fiber bundle has a total diameter of 1000 µm at the tip and a numerical aperture of 0.22. The light guide is connected to a box that houses both the optical units and the electrical unit. Fluorophores of interest are fluorescent‐active proteins (EX280/EM340), NADH (EX365/EM450), and flavin (EX450/EM525). Additionally, scattered light can be measured at 830 nm in order to obtain information about OD. The optical units are separated in an excitation and a detection unit. For the optical part, the surrounding light is totally eliminated by integration into an opaque polyoxymethylene enclosure. Monochromatic, high power LEDs are used for excitation and are installed on a wheel. The LED wheel is controlled by a stepper motor with a resolution of 1.8°. The distance between light guide and LED is 0.5 mm, so that maximum of light is transferred into the light guide. When selecting the LEDs, both the wavelength and the narrowest possible bandwidth were taken into account. LEDs were used as high power surface‐mount device LEDs (Roithner Lasertechnik GmbH, Austria) and are listed in Figure 1.

The detection unit consists of a sensitive photodiode (Luna Optoelectronics, USA) with optical filters (Edmund Optics Inc., UK) on a wheel in front. The wavelengths of the filters were chosen with focus on small bandwidth, high OD, and wavelength of the fluorophores and are listed in Figure 1. These filters can be changed by a stepper motor with a resolution of 1.8°. Empty spaces remain on the wheel to allow the addition of more filters if required. The distance between photodiode, filter, and light guide is kept at a minimum of 0.5 mm in order to keep the intensity loss low. In total, 16 combinations could be used for analysis, but only four are meaningful for the application of biofilm monitoring.

The positions of LEDs and filters were controlled after 100 measurements. For this purpose, a reference with a light trap was installed. The whole sensor is controlled by a user‐made software with programs for measurement of all fluorophores.

### 2.2 Calibration curves of BSA, riboflavin, and NADH

Characterization of the sensor was performed with reference solutions of NADH, riboflavin as reference for flavin, and BSA as reference for the protein channel. The fluorophores BSA (Sigma–Aldrich, St. Louis, USA), NADH, and riboflavin (Sigma–Aldrich) were dissolved in PBS (pH 7.5). Concentrations can be found in Section 3. The samples were measured at the respective wavelengths in black microtiter plates (Thermo Fisher Scientific, Massachusetts, USA) for excluding the surrounding light. All measurements were performed in triplicates and the linear measuring range was determined. As reference, a second measurement with the same samples was performed using a fluorescence spectrometer (Hitachi F‐7000, Japan).

An additional calibration was performed in order to test the specificity of the sensor. For this test, the signal of the fluorophore assayed was measured alongside the signal of the other channels.

### 2.3 Selectivity test

To demonstrate the selectivity of the sensor, 19 solutions with linear independent concentration levels of the biological fluorophores were prepared in PBS (pH 7.5). The scheme for the concentration levels was generated using the design of experiments according to Graf et al. [35]. This approach allows the minimization of the number of experiments and maximizes the information content [36]. Fluorescence measurements for each fluorophore were done in black microtiter plates (Thermo Fisher Scientific) to exclude surrounding light effects. The evaluation of the measurement data was carried out by means of multilinear regression with three variables using the software “The Unscrambler X 10.1” (CAMO Software, Norway).

### 2.4 Comparison between immobilized riboflavin and riboflavin in solution

Riboflavin as model analyte for biogenic fluorophores was immobilized in agarose in order to test the developed sensor for measurements in gel‐like structures. A 4 × 10^−5^ mol/L riboflavin solution was prepared in double‐distilled H_2_O and was mixed with several concentrations of alginate (1, 2, 3, and 4% w/v; Geniesser Depot, Germany). A gel of each riboflavin–alginate solution was made using 100 mM CaCl_2_ as an immobilizing reagent. For this purpose, the riboflavin–alginate solution was coated with a CaCl_2_ soaked filter paper for 1 h. Nonimmobilized riboflavin was used as the reference solution. After immobilization, each gel was placed in a black microtiter plate (Thermo Fisher Scientific) and measurements with the fluorescence sensor were performed in each gel and the reference solution. For gel measurements of riboflavin, the sensor was positioned 5 mm deep in the gel and also directly on the surface.

### 2.5 Stability test

The long‐term stability of the electrical components and the measured signal was tested for the sensor. For the evaluation of signal stability, the scattered light of the various LEDs was measured using a measuring chamber. A white filter paper was used for reflection on the bottom of the chamber. The LEDs were coupled into the light guide one after the other and the backscattered light was measured at the photodiode. Both the reliability of the positioning of the components by the stepper motors and the accuracy of the reference run were tested. The reference run is a component of the system that is intended to ensure that the LEDs and the optical filters are positioned precisely even after repeated changes. A reference run is performed every 100 measurements. The whole measurement was done over 48 h with a measuring time period of 10 min. The LED with a wavelength of 280 nm could not be used in this experiment due to the sensitivity restriction of the photodiode at this wavelength.

### 2.6 Induced change in metabolism

As a proof of principle that the developed fluorescence sensor can assay metabolic changes of cultures, an aeration experiment in a *Saccharomyces cerevisiae* culture was performed. Forty two grams bakery yeast from the local supermarket (UNIFERM GmbH & Co. KG, Germany) was washed three times in 0.9% w/v NaCl and centrifuged (10 000 rpm, 10 min) for separating *S. cerevisiae* from other components of the bakery yeast. The pellet was resuspended in Schatzmann buffer (4.5 g/L (NH_4_)_2_SO_4_, 1.9 g/L (NH_4_)_2_HPO_4_, 0.9 g/L KCl, pH 4, OD_600nm_ 20) and 10 g/L glucose was added. The culture was aerated alternately with pure oxygen and pure nitrogen over a micro‐sparger in a round flask at room temperature. In addition, the culture was stirred with a magnetic stirrer to homogenize the contents. The fluorescence sensor and an optical pO_2_ sensor (PreSens GmbH, Germany) were positioned directly in the culture to measure NADH (EX365/EM450) and pO_2_. Aeration was changed from oxygen to nitrogen in a time period of 10 min and switched back to oxygen with a time period of 20 min.

The experiment was repeated for detection of change in metabolism in a biofilm‐like structure. Instead of Schatzmann‐buffer, a 0.1 M citrate‐buffer (pH 4) with 15 g/L glucose was used as medium for the yeast. Bakery yeast was washed, centrifuged, and prepared in citrate‐buffer to an OD_600nm_ of 40 as described above. Afterwards, the yeast was immobilized in 3% w/v sodium alginate (Geniesser Depot, Germany). The buffer–yeast suspension was mixed with the sodium alginate in a ratio of 1:2 and poured onto a filter paper soaked with 100 mM CaCl_2_. Immobilization was performed for 1 h at 4°C.

After immobilization, the gel was fixed in a round‐bottom flask with an additive manufactured mounting. A micro‐sparger for aeration was positioned under the mounting in the round‐bottom flask. In addition, the culture was stirred with a magnetic stirrer to homogenize the contents. The fluorescence sensor was positioned directly in the gel and a pO_2_ sensor (PreSens GmbH) was placed at the surface of the gel. An aeration switch between pure oxygen and pure nitrogen was performed after achieving signal constancy.

### 2.7 Wastewater‐based biofilm

**FIGURE 2:**
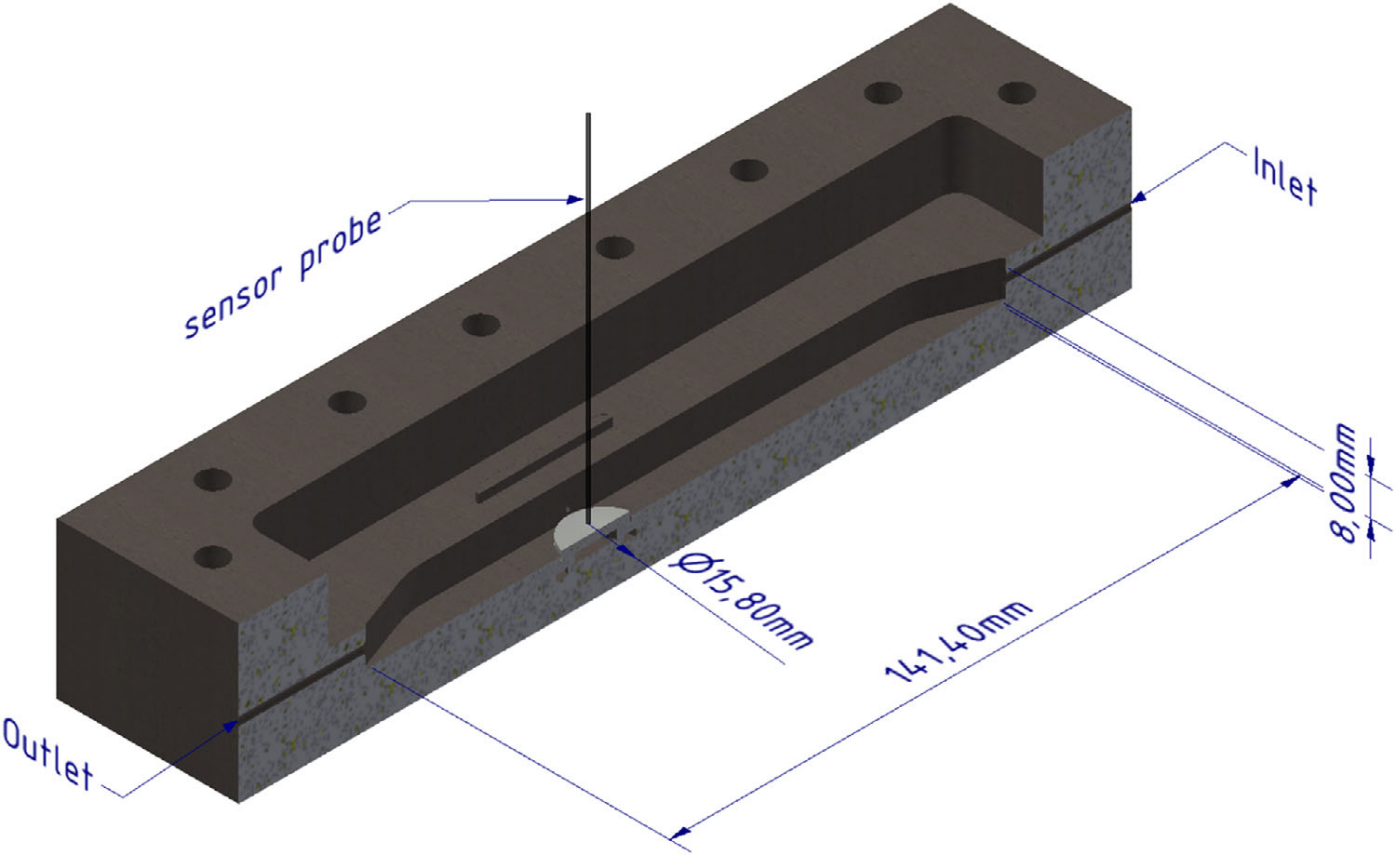
3D design of the used flow cell (cross section). The light guide of the sensor can be positioned through the lid close to the growth surface. Dimensions of the flow cell are presented. Lid for closing the flow cell is not shown for a better overview

Colonies from a kitchen sink were picked as inoculum and suspended in a defined media (1.7 g/L yeast nitrogen base, 2.69 g/L NaH_2_PO_4_∙H_2_O, 4.33 g/L Na_2_HPO_4_, 0.31 g/L NH_4_Cl, 0.13 g/L KCl, 10 g/L glucose, and pH 7.5) and cultivated in a flow chamber (Figure 2; construction drawing prepared and provided by the Institute for Chemical and Thermal Process Engineering, TU Braunschweig). The flow chamber consists of a graphite plate as a growth surface, which is flushed over by media. The sensor probe can be firmly positioned close to the growth surface through the lid of the chamber. A simple O‐ring on the sensor enables an exact and reproducible positioning of the sensor in the flow cell.

The media was circulated with a flow velocity of 2.5 mL/min^−1^ at room temperature. The fluorescence sensor was positioned in the flow chamber close to the graphite plate (*r* = 7.9 mm, *h* = 2 mm), which was used as a growth surface due to its porosity. Media was changed after reaching a constant signal of NADH, flavin, and proteins. For this purpose, chamber contents were pumped out and the storage bottle was replaced with fresh media. After changing the media, the measurement was continued for 1 h to ensure that the previous signals originated in the biofilm. At the end of the experiment, the graphite plate was positioned under a 3D microscope (VHX Keyence, Japan) to confirm the biofilm development. This experiment was performed to show that the sensor can be used for online monitoring of a biofilm in a reactor normally used for a microbial fuel cell.

## 3 RESULTS AND DISCUSSION

### 3.1 Sensitivity and selectivity

**FIGURE 3:**
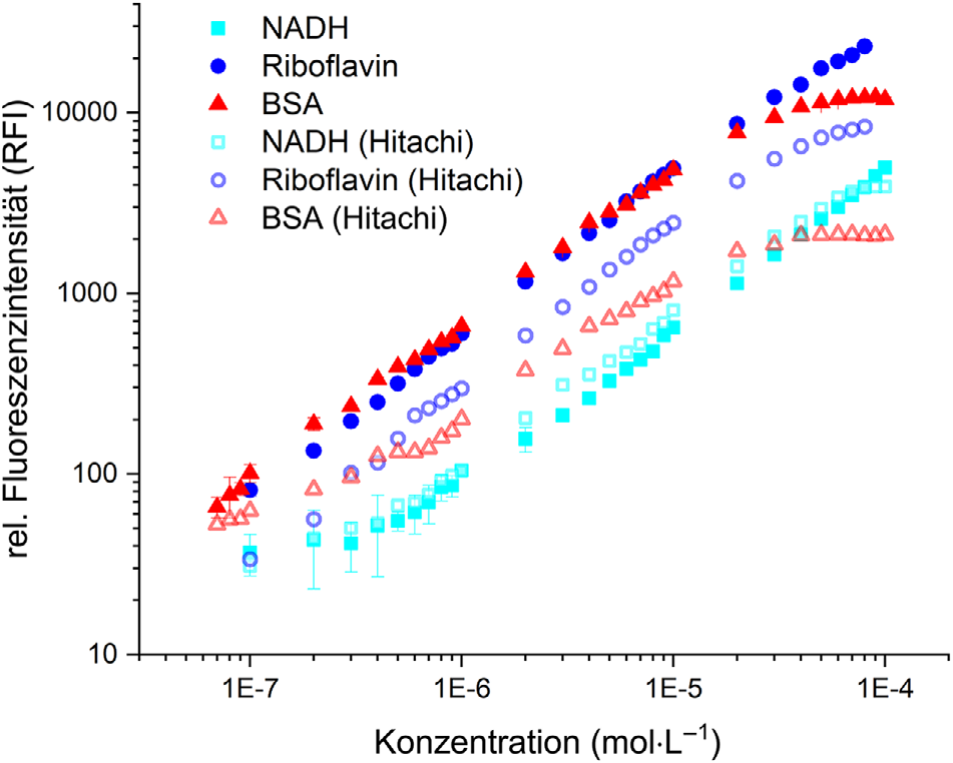
Calibration curves of BSA (EX280/EM340), NADH (EX365/EM450), and riboflavin (EX450/EM525). Measurements were carried out by using the developed fluorescence sensor and a fluorescence spectrometer (Hitachi) as reference

Figure 3 shows the results of the calibration measurements. A linear measuring range could be determined for all measured fluorophores. For NADH a linear measuring range between 5 × 10^−7^ and 1 × 10^−4^ mol/L (*R* ^2 ^= 0.99) could be established. As only NADH is fluorescently active and NAD^+^ does not exhibit fluorescence, the determined linear range reflects only NADH levels. Riboflavin has shown higher fluorescence intensities but a similar linear range between 1 × 10^−7^ and 8 × 10^−5^ mol/L(*R* ^2 ^= 0.99). BSA was used as a reference for determining the linear range of proteins. At high protein concentrations, a saturation of fluorescence intensity was observed, which could be attributed to the inner filter effect [37]. The linear range for proteins is between 7 × 10^−8^ and 2 × 10^−5^ mol/L(*R* ^2 ^= 0.98). The SD of the measurements is only significant at intensities <100 relative fluorescence intensity (RFI).

Furthermore, the results were confirmed using a reference fluorescence spectrometer (Hitachi F‐7000, Japan). The same linear range could be determined for all three components (*R* ^2^_NADH _= 0.99, *R* ^2^_riboflavin _= 0.99, and *R* ^2^_BSA _= 0.98) with the Hitachi instrument as well.

The linear range of the fluorescence sensor developed in this work is comparable to the measuring range determined by König et al., who successfully used a similar fluorescence sensor, but with a larger tip diameter, for monitoring cultivations of suspension cells in bioreactors [34].

In bacterial cells, NADH concentrations in the range of 10^−6^ to 10^−3^ mol/L are expected [38, 39, 40, 41, 42]. Bennett et al. determined NADH concentrations of around 10^−5 ^mol/L and protein and flavin concentrations of about 10^−5^ mol/L for planktonic, aerobic grown *Escherichia coli* in the exponential growth phase (OD_650_ of 0.35) [43]. In cells growing as a biofilm, Dietrich et al. determined NADH/NAD^+^ concentrations in the range of 4 × 10^−6^ mol/L to 13 × 10^−6^ mol/L for *Pseudomonas aeruginosa* [44]. Flavin concentrations of up to 7 × 10^−7^ mol/L and lower were reported in biofilms of *Shewanella oneidensis* [45]. The determined linear ranges of the developed sensor are thus well within expected biological levels.

**FIGURE 4:**
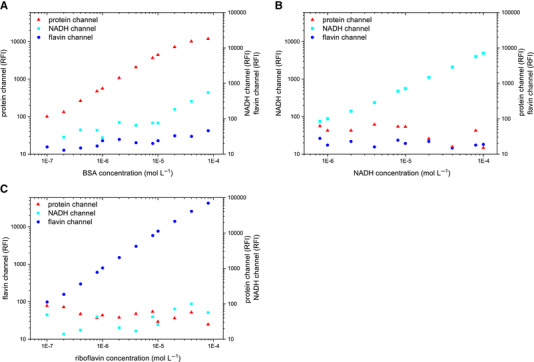
Determination of channel specificity of (A) protein channel, (B) NADH channel, and (C) flavin channel. The relative fluorescence intensities of the other channels are displayed in each specific channel shown

When using optical sensors for analyzing biofilms, it is important to know whether the sensor performs sensitive, specific, and selective measurements. Therefore, a second calibration was carried out by measuring the fluorescence intensity in reference solutions of BSA, NADH, and riboflavin simultaneously. This experiment evaluates if the measurement of a specific channel is selective. It could be shown that the individual channels measure in a specific, as well as sensitive manner (Figure 4). Fluorescence intensities were only measured for channels corresponding to the reference solution being tested. Signals of the other channels were <100 RFI, which is in the range of background noise. Only in highly concentrated samples of BSA, a NADH signal could be also measured. This can be explained by the inner filter effect [37] due to high BSA concentrations.

In the next step, the selectivity of the sensor was determined by investigating the influence of the biogenic fluorophores on each other. As the fluorescence spectra overlap, riboflavin can also be excited when measuring NADH at 450 nm. Whether this cascade impacts the measurement is decisive for monitoring bioprocesses, because all fluorophores will be present in parallel. The specific concentrations used in the selectivity experiment were determined by using principle component analysis. This ensures a reliable statement despite minimal measuring points.

**FIGURE 5:**
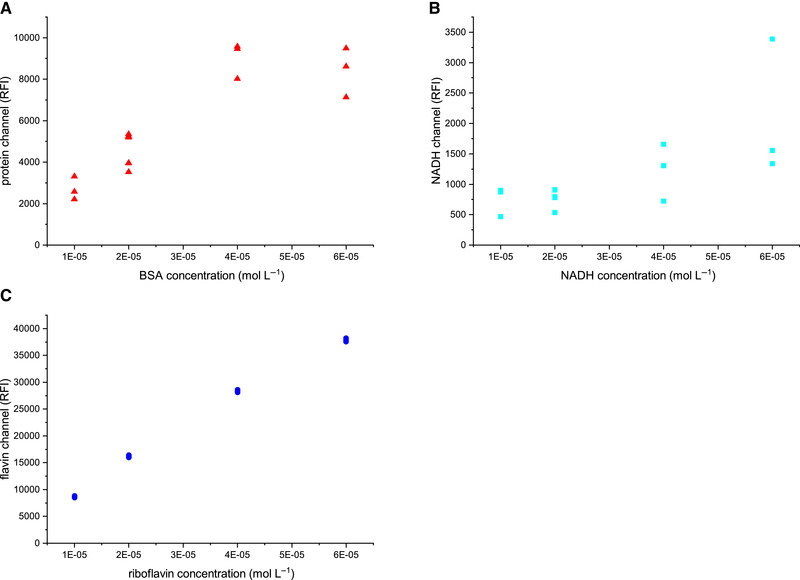
Determination of selectivity. Correlation of fluorescence intensities of proteins (A), NADH (B), and flavins (C) are shown. The measurements were performed in solution of different concentrations of BSA, NADH, and riboflavin

The evaluation showed that both the measurement of proteins and flavins is selective and not influenced by other fluorophores (Figure 5). Here, the measured fluorescence intensities correlate directly with the concentrations used. The protein channel shows a comparatively large scatter of measured values that is caused by the amplification of the signal. However, the measurement of NADH is influenced by the presence of other fluorophores. Therefore, a multilinear regression with three variables was performed for the NADH channel, which showed that the measurement is negatively influenced by proteins and flavins. This result was to be expected due to the overlapping fluorescence spectra. For the determination of the NADH concentration (*y*_NADH concentration_), the relative fluorescence intensities (*x*_fluorescence channel_) have to be subjected according to the Equation (1), which was determined with *R* ^2 ^= 0.95. Based on the regression coefficients, it can be assumed that under these experimental conditions BSA has a negative and riboflavin a positive influence on the NADH signal.

(1) $$\begin{array}{ccc} y_{\mathrm{NADH}\;\mathrm{concentration}} & = & -8.55\times 10^{-10}x_{\mathrm{protein}}\\ & & +\;3.52\times 10^{-8}x_{\mathrm{NADH}}\\ & & +\;8.56\times 10^{-10}x_{\mathrm{flavin}}-1.67 \end{array}$$

Overall, it can be said that the fluorescence sensor can selectively determine the desired fluorophores. When measuring NADH, it is very important to determine both proteins and flavins in order to obtain the truly selective readings. In biological processes, however, the fluorescence signal is also influenced by media components. For a model that allows the direct calculation of concentration from measured fluorescence intensities, additional influencing variables must be determined and taken into account.

### 3.2 Comparison between immobilized riboflavin and riboflavin in solution

**FIGURE 6:**
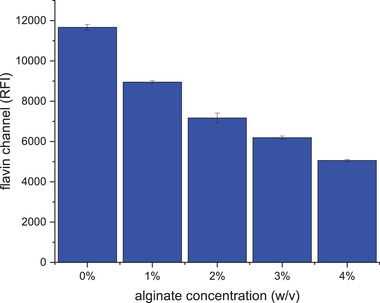
Comparison between immobilized and suspended riboflavin. Riboflavin was immobilized in several concentrations of alginate. Fluorescence measurements were performed in immobilized riboflavin as well as in suspended riboflavin (0% w/v alginate concentration)

In order to start transferring from measuring in solution to the intended application as a biofilm sensor, an experiment measuring immobilized and free riboflavin in solution was performed. Figure 6 shows the result of the measurement in suspended and immobilized riboflavin. Due to the increasing alginate density, riboflavin can no longer be excited consistently. This leads to a decreasing fluorescence intensity with increasing alginate concentration.

Of special interest is the influence of 1.5% w/v alginate on the fluorescence measurement because this concentration was used to immobilize the bakery yeast in further experiments. Compared to suspended riboflavin, the fluorescence intensity of immobilized riboflavin is 32% lower.

### 3.3 Stability tests

**FIGURE 7:**
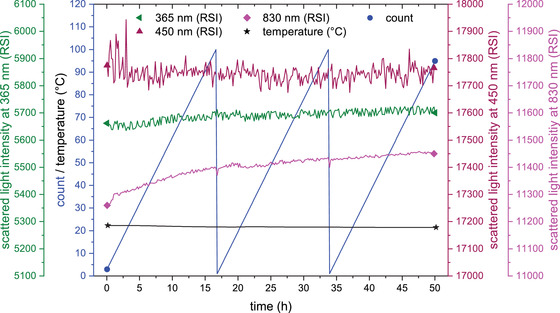
Stability analysis of the fluorescence sensor. Scattered light was measured over 50 h. Raw signals are presented, together with reference count and temperature. The count data represents the number of measurement runs. After 100 measurement runs, a reference run is performed and the counter is reset to zero

The measurement of the long‐term stability (Figure 7) shows that the system functions in a very stable manner. The measurement noise of the scattered light of the 365 nm and the 450 nm LED is 2%; the noise of the 830 nm LED <1%. With the 830 nm LED, however, the signal increases by 2% over the entire measurement period. Later experiments showed that the signal does not increase any further over a longer period of time with the same experimental setup.

### 3.4 Analysis of yeast suspension culture

In order to analyze whether the fluorescence sensor is also suitable for the direct assay of a biological system especially in regard to metabolic changes, a culture of *S. cerevisiae* was monitored with the sensor. A metabolic change was induced that involves switching between aerobic and anaerobic conditions [46]. In the presence of oxygen, NADH is oxidized to NAD^+^ as a coenzyme in Complex I of the cell respiratory chain. If, however, anaerobic conditions are present, NADH cannot be oxidized in the cell respiratory chain and will be directed in anaerobic fermentation. Compared to the aerobic metabolism, NADH concentration is significantly lower in anaerobic processes. This change in NADH concentration can be monitored with the fluorescence sensor.

**FIGURE 8:**
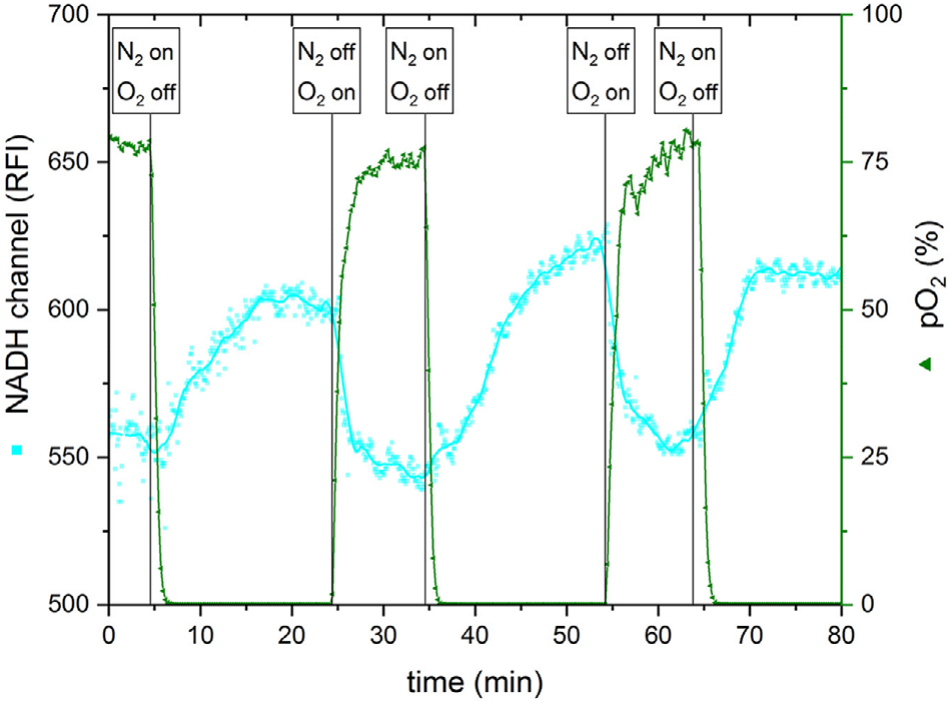
Fluorescence intensity of NADH during induced change of metabolism in a suspension culture of *S. cerevisiae*. For the induced change in metabolism a switch between oxygen and nitrogen is performed. During the whole experiment the fluorescence intensity of NADH and pO_2_ was measured

The results of this experiment are shown in Figure 8. It could be demonstrated that the expected metabolic switch occurs and can be detected with the sensor. After switching aeration from pure oxygen to pure nitrogen, the signal of the NADH channel increased up to 600 RFI. After changing the system back to oxygen aeration, the NADH signal decreased rapidly. The process could be repeated several times with similar results. Note that 100% aeration is not reached due to the experimental setup that constitutes an open system. In conclusion, it could be shown that the developed sensor can be used for measurements in suspended cell systems.

### 3.5 Analysis of immobilized yeast culture

**FIGURE 9:**
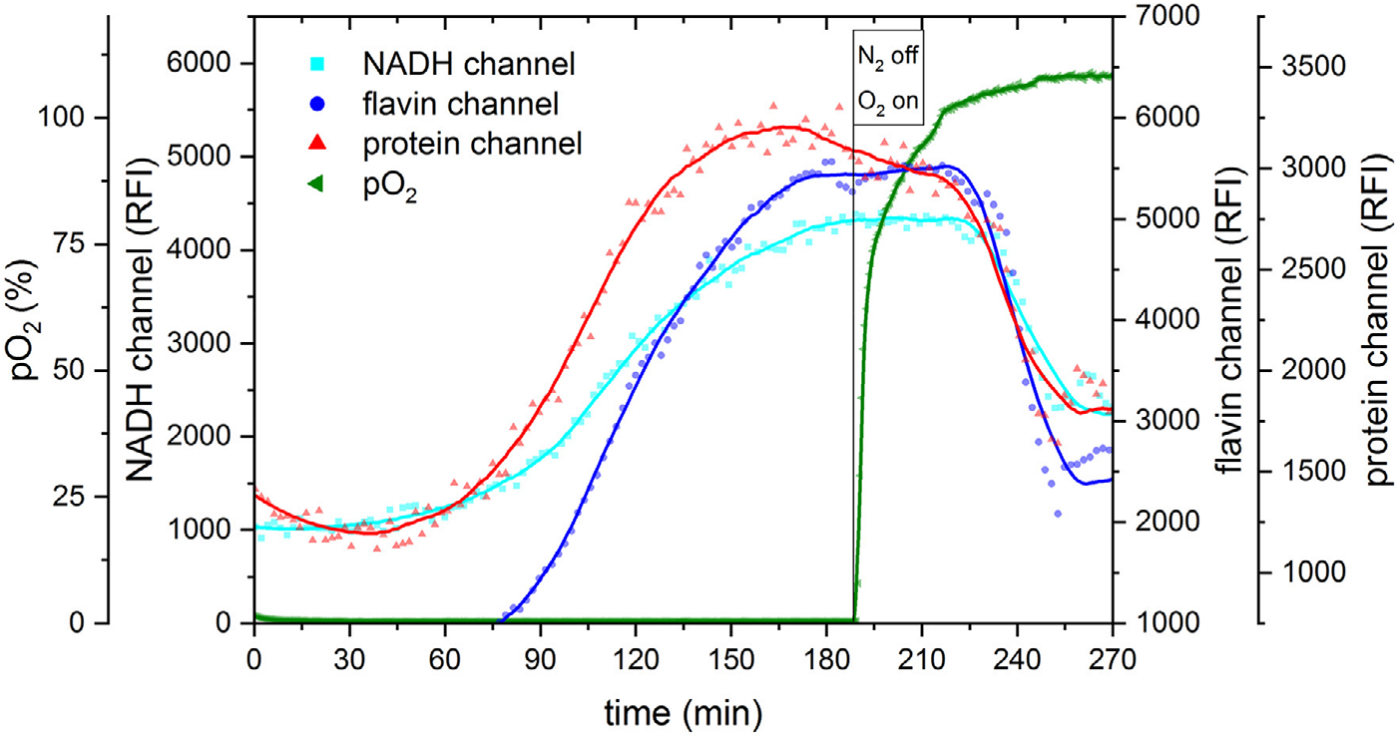
Monitoring of a metabolic switch in an immobilized yeast culture. Shown are fluorescence intensities of NADH, flavin, and proteins. The pO_2_ is presented as well. Bakery yeast was washed and afterwards immobilized in an alginate gel. The fluorescence sensor was positioned in the gel. After 190 min, nitrogen aeration was turned off and an aeration with oxygen was performed

In the next step, it was tested whether the developed sensor is suitable for the direct assay of a biofilm‐like structure. For this purpose, a culture of *S. cerevisiae* was immobilized and a metabolism change was induced like in the yeast suspension culture previously. Due to immobilization and the resulting diffusion limitation [47], a longer time lapse was expected until a change in metabolism occurs. From literature, it is known that the diffusion coefficient in an alginate gel depends on the cell concentration in the gel. Sato and Toda determined a diffusion coefficient of oxygen in a 2% alginate gel of 0.070 cm^2^/h at 30°C, which is about 70% of that in water [47, 48, 49]. Compared to the previous experiments with suspended yeast, a metabolism change could be detected only after 90 min (Figure 9). In addition to NADH, the fluorescence intensities of flavins and proteins were measured too [6]. All measured components increased in their fluorescence intensity, which indicated not only a change in metabolism but also a change in the metabolic growth of the immobilized cells. Before starting aeration, the cells were immobilized in the gel for 1 h. During this time, anaerobic conditions could have occurred in the gel and the cells probably switched to anaerobic fermentation that led to cell growth.

After constant values in fluorescence intensity were reached, oxygen aeration was started. This led to a decrease in fluorescence intensity in all channels with increasing pO_2_. As oxidized flavins are fluorescent active, it was expected that the fluorescence intensity should increase. The decreasing fluorescence intensities indicate that an induced change in metabolism did not occur as expected. However, as growth in the immobilized culture occurred under anaerobic conditions before starting the experiment, the observed changes indicate a reaction after the aeration change. Compared to the previous experiment in suspension, it was not possible to perform several aeration cycles, because the gel started to disintegrate. Nevertheless, it could be shown that the developed fluorescence sensor is also able to detect metabolic processes in gel‐like structures.

### 3.6 Analysis of a wastewater‐based biofilm

**FIGURE 10:**
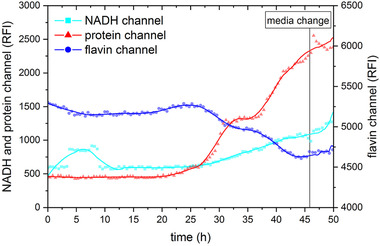
Fluorescence intensities of NADH, flavin, and protein during biofilm growth. After 46 h, the media was pumped out and fresh media pumped in to confirm that fluorescence is based on biofilm growth. All signals were smoothed using a Savitzky–Golay filter

A wastewater‐based biofilm — as a model for a biofilm used in a microbial fuel cell — was grown in a special flow cell. The developed sensor was used to monitor the biofilm. The fluorescence intensity of proteins, NADH, and flavins was measured during the whole process and the raw signals obtained were smoothed using a Savitzky–Golay filter. Figure 10 shows the measured fluorescence intensities during the biofilm development. The fluorescence signals remain constant during the first 25 h of cultivation. It should be mentioned that a signal drift in the NADH channel could be detected during the first 10 h. This drift can be explained by a positioning error of the LED during the reference run. As the reference run is repeated after 100 measurements and the signal returns to the previous value, the observed drift in the NADH signal can be interpreted as an outlier.

After 25 h, the signals of all fluorescence channels were changing: the NADH and the protein signal increased while the flavin signal decreased in intensity. The behavior of NADH and protein signal can be explained by the adhesion of cells and the formation of a biofilm. The decrease in flavin signal associated with biofilm growth was not expected and has to be analyzed in further experiments. After a cultivation time of 46 h, when the NADH and flavin signals were constant, the cultivation medium was changed to check if the measured signal was based on planktonic cells or on the formation of a biofilm structure. If the signal is based on planktonic cells, the fluorescence intensity should return to initial values after the media change. In this experiment, constant fluorescence intensities could be measured, which strongly indicates biofilm formation.

**FIGURE 11:**
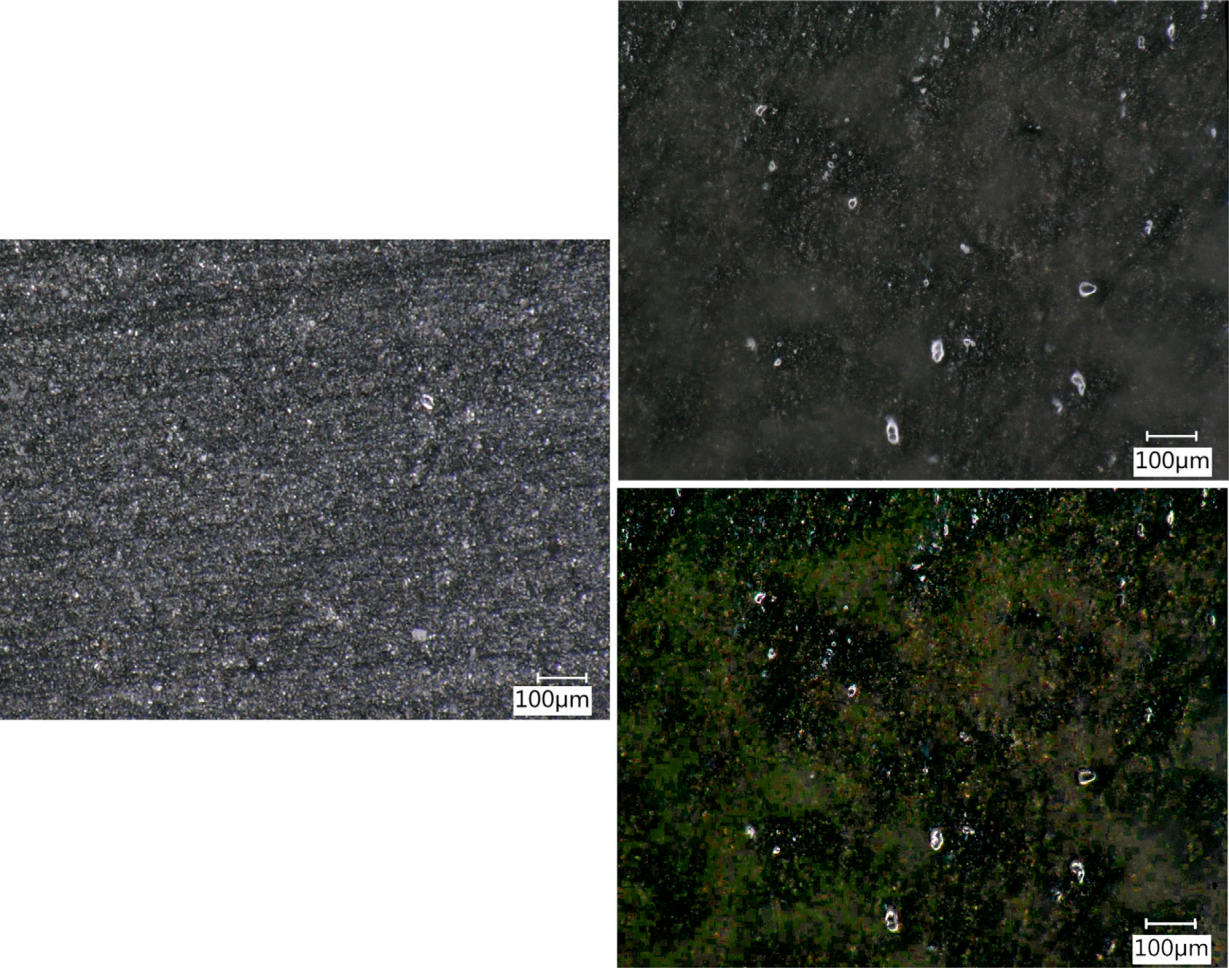
Microscopic images of the graphite plate before (left) and after (right) biofilm cultivation. Top right: After cultivation, a 3D structure could be seen using a 3D microscope. Bottom right: The same image is presented as above. For clarification, image processing was performed by changing contrast and brightness. The green structures in this image are the biofilm that could be seen under the microscope. The growth of this structure was also measured by the developed fluorescence sensor. Magnification: 150×; angle of focusing: 22°

Additionally, the graphite plate was taken out of the flow chamber and investigated under a 3D‐microscope. The microscopic images of the graphite plate before and after the cultivation are presented in Figure 11. After cultivation, a slimy structure could be seen on the graphite plate on visual inspection and also by microscope analysis. Due to the inhomogeneous structure, it was not possible to obtain an image with higher resolution. It could be shown that the developed sensor could monitor the development of a biofilm.

## 4 CONCLUDING REMARKS

A new, compact fluorescence sensor with a small sensor tip unit for biofilms was developed. It is characterized by its fiber sensor tip with a small diameter of 1 mm, its mobility, and its high stability during long‐term measurements. Furthermore, a linear measuring range could be determined for proteins, NADH, and flavins, which is comparable to other fluorescence‐based sensors like the Hitachi fluorescence spectrophotometer and the sensor developed by König et al. It could be shown that the sensor measures very sensitively, specifically, and selectively for the individual fluorophores. In combination with the small diameter that allows local measurements, the sensor will be able to measure with a high resolution in a biofilm.

Due to the thin light guide and the goal of coupling in several wavelengths, an LED and a filter wheel were used as mobile components. It could be shown that positioning errors of the wheels can be compensated by the reference run. The reference run will be optimized in further experiments.

Experiments have shown that the sensor can measure both in solution and in gel‐like structures. In addition, a measurement in a wastewater‐based biofilm was performed successfully. Thus, the sensor can be used for different applications. One possible application is the use in a microbial fuel cell, where a wastewater‐based biofilm is grown on the electrode surface for the generation of electrical current. Here, the analysis of biofilm development is of particular importance to ensure a constant current. The use of an online measuring method could increase the efficiency of the microbial fuel cell due to better monitoring. Additional optical filters can be integrated into the sensor and thus extend its range of application.

The next development steps planned include a characterization of the scattered light channel. Furthermore, the calibration measurements will be repeated in cultivation media in order to develop a correlation between signal and fluorophore concentration. A micromanipulator will be added in order to achieve a measurement at several positions in the biofilm and at its environment.

## CONFLICT OF INTEREST

The authors have declared no conflict of interest.
